# Use of genetically modified lactic acid bacteria and bifidobacteria as live delivery vectors for human and animal health

**DOI:** 10.1080/19490976.2022.2110821

**Published:** 2022-08-12

**Authors:** Romina Levit, Naima G. Cortes-Perez, Alejandra de Moreno de Leblanc, Jade Loiseau, Anne Aucouturier, Philippe Langella, Jean Guy LeBlanc, Luis G. Bermúdez-Humarán

**Affiliations:** a Centro de Referencia para Lactobacilos (CERELA-CONICET), Chacabuco 145, (T4000ILC) San Miguel de Tucumán, Tucumán, Argentina; b Université Paris-Saclay, INRAE, AgroParisTech, UMR 0496, 78350 Jouy-en-Josas, France; c Micalis Institute, Université Paris-Saclay, INRAE, AgroParisTech, 78350 Jouy-en-Josas, France

**Keywords:** Lactic acid bacteria, *lactococcus lactis*, *lactobacillus* spp, bifidobacteria, live delivery vectors, mucosal vaccines

## Abstract

There is now strong evidence to support the interest in using lactic acid bacteria (LAB)in particular, strains of lactococci and lactobacilli, as well as bifidobacteria, for the development of new live vectors for human and animal health purposes. LAB are Gram-positive bacteria that have been used for millennia in the production of fermented foods. In addition, numerous studies have shown that genetically modified LAB and bifodobacteria can induce a systemic and mucosal immune response against certain antigens when administered mucosally. They are therefore good candidates for the development of new mucosal delivery strategies and are attractive alternatives to vaccines based on attenuated pathogenic bacteria whose use presents health risks. This article reviews the most recent research and advances in the use of LAB and bifidobacteria as live delivery vectors for human and animal health.

## Introduction

Lactic acid bacteria (LAB) are Gram-positive bacteria that produce lactic acid as the main end-product of glucose fermentation. LAB are classified according to their phenotypic and metabolic characteristics such as cellular morphology, growth conditions (as temperature and sugar utilization) and the production of substances to inhibit the proliferation of other microorganisms during the fermentation process. LAB are members of the phylum Firmicutes, and order Lactobacillales^1^. Concerning bifidobacteria, even if some of their morphological and metabolic characteristics (as the production of lactic acid during fermentation), led them to be classified initially as members of LAB, currently are recognized as part of an independent genus, *Bifidobacterium*, that belongs to Actinobacteria phylum and the Bifidobacteriales order.^2,3^ However, it would not be surprising that within a few years this classification will face considerable changes. Indeed, as Hugenholtz *et al*. point out in their recent review article, the increasing number of genomic sequences derived from unculturable prokaryotes makes taxonomic classification a major challenge for consensus and adaptation.^4^ An important number of species belonging to both LAB and Bifidobacterium are widely used in industrial food fermentation processes and some genera are inhabitants of the intestinal microbiota.^5,6^ In addition, some of these species, when ingested in appropriate quantities, can survive passage through the digestive tract and exert different beneficial actions (*e.g*., improving fiber digestion, stimulating the immune system, and preventing or treating diarrhea); moreover, as mentioned above they are classified as GRAS and QPS microorganisms and several strains are considered as probiotics. Probiotics have been defined by the World Health Organization (WHO) in 2011, but a group of experts reexamined the concept in 2014 to reach a consensus definition as follows: *“live microorganisms that, when administered in adequate amounts, confer a health benefit to the host”*.^7^

For all these reasons, the use of LAB and Bifidobacterium as vectors for biologically active molecules is a strategy that has aroused great interest, notably on the development of new live mucosal delivery vectors. In addition, mucosal vectors (e.g., administered orally, intranasally, vaginally, etc.), are more convenient than classical systemic routes of administration, because they are easier to administer and relatively cheaper to produce. For instance, the human mucosa (including the gastrointestinal, respiratory, and urogenital tracts) represents a major contact surface estimated at approximately 400 m2,^2,8^ besides containing a highly developed immune system: the Mucosa Associated Lymphoid Tissue (MALT) consisting of about 80% of the body’s immune cells and considered the most important lymphoid system in human.^9–11^

## LAB and Bifidobacterium as new live delivery vectors

**Figure 1.**
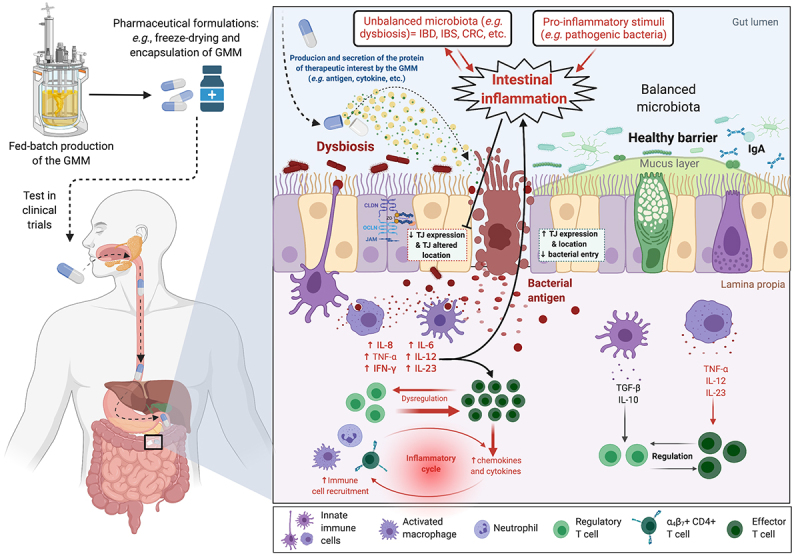
Schematic overview of the production (*e.g*., fed-batch production) of a genetically modified microorganism (GMM) to deliver a therapeutic molecule. Example of the *in situ* production and of a protein with anti-inflammatory properties by a GM *L. lactis* strain in the context of intestinal inflammation.

The constant need to develop safer, easier to administer and cheaper vectors, such as vaccines, has led to intensive research on the possible use of live genetically modified microorganisms (GMM) as carriers of proteins of interest, such as protective antigens, especially for *in situ* administration. Attention has therefore turned to the use of Gram-positive and commensal LAB as protein delivery vectors. In this sense, the genetic and molecular studies carried out in the last years, mainly in *Lactoccoccus lactis* and some species of lactobacilli, have demonstrated that these bacteria can be used for this purpose in the prevention and treatment of diseases (Figure 1). Different studies have revealed the potential of these bacteria to be used as drug carriers or to produce therapeutic molecules due to their intrinsic (adjuvant and immunomodulatory) natural properties.^12^ These bacteria are also of interest from a technological point of view because some strains are resistant to low pH and therefore can survive the passage through the gastrointestinal tract and can adhere to the intestinal epithelium which makes them interesting for oral vaccine. Even today, other strategies have been developed to preserve bacterial cells during their passage through the stomach, such as encapsulation in microparticles or liposomes, which further improves their adhesion to the mucosa. Another alternative is the use of liquid carriers with protective properties such as trehalose.^13^ In addition, they do not need to be stored at low temperatures because they can be lyophilized.^14^ Their complete safety due to generally recognized as safe (GRAS status, FDA, USA)^15^ “GRAS” status and with qualified presumption of safety (QPS, EFSA, Europe), combined with the ability of some of them to colonize external body cavities, makes

LAB, in particular lactobacilli, the candidates of choice to deliver vaccine antigens to the mucosa. These bacteria have the additional advantage of being easily administered orally or locally. Thus, the use of LAB as antigen vectors is a safer and less expensive strategy. In addition, these bacteria have already been used to express and deliver several proteins of medical interest.

## Genetic engineering of LAB and Bifidobacteria strains Use of *Lactococcus lactis* as a live delivery vector

*Lactis* is a Gram-positive bacterium widely used in the manufacture of dairy products, especially cheese. In addition, this bacterium can synthesize bacteriocins,^16^ which generally prevents the development of undesirable microorganisms in dairy products and helps to preserve the hygienic quality of the products. The numerous studies carried out on this bacterium have made it possible to characterize essential genes or genes of technological interest (genes involved in metabolism, stress resistance, growth, etc.), and to elucidate their expression mechanisms.^17^ Increasingly efficient study tools and techniques have been developed such as mutagenesis and chromosomal integration systems for genes of interest, cloning and constitutive or inducible expression vectors, systems for targeting heterologous proteins in different compartments of the bacterium.^18,19^ In addition, the genomes of *L. lactis* subsp. *lactis* IL1403 and *cremoris* MG1363 have been sequenced.^20,21^ Thanks to this work, *L. lactis* is considered as the model LAB and is one of the best characterized bacteria along with *E. coli* and *Bacillus subtilis*. The development of knowledge on its capacities to produce and secret heterologous proteins makes it a good candidate for the secretion of proteins of therapeutic interest.

*L. lactis* has been widely studied and manipulated in recent years for the production of heterologous proteins such as several viral or bacterial antigens, as well as biologically active molecules (cytokines, hormones, etc.).^19,22^ In this context, our team has developed several tools to optimize the production of heterologous proteins in this bacterium, which we describe in the next paragraphs.

## Vectors for heterologous protein expression in *L. lactis*

**Figure 2.**
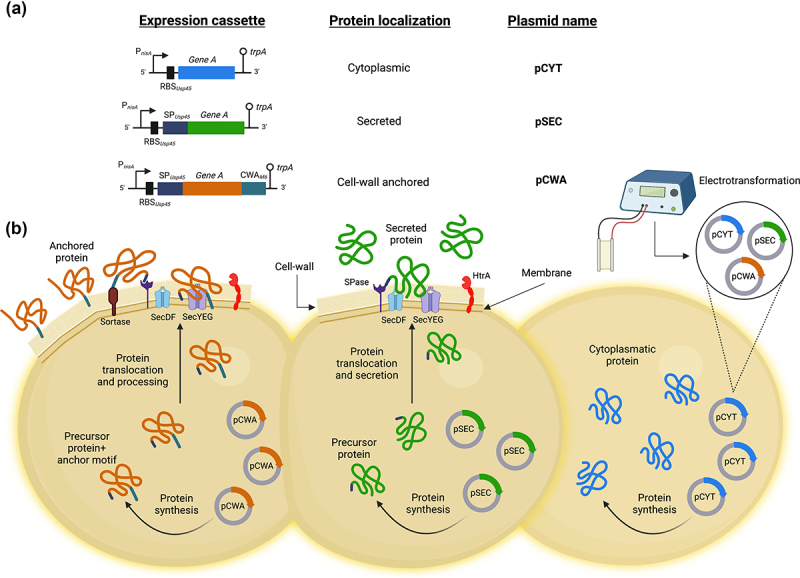
Family of vectors that allow controlled expression and export of proteins in *L. lactis*. **(A)** Schematic structures of different expression cassettes (left) under the control of the lactococcal P*_nisA_* promoter for the indicated specific bacterial cellular localization and carried by the specified plasmids. For details of the plasmid constructions see the text. Stems topped with circles indicate the tryptophan transcriptional terminator (*trpA*). Not to scale. **(B)** Graphical representation on the production of the desired protein by using the plasmid indicated for the different bacterial localization of interest in *L. lactis*. **pCYT**: to obtain the expression of a protein in the cytoplasm, the gene of interest is fused only to the *P_nisA_* promoter. **pSEC**: in which the secretion pathway used is the Sec-dependent pathway. It recognizes proteins synthesized with an N-terminal signal peptide (SP) and ensures their export and translocation. It is worth highlighting that the nature of the SP used to secrete a protein can greatly influence the secretory efficiency of the protein. Thus, one of the most efficient SP for secreting heterologous proteins in *L. lactis* is that of the Usp45 protein (*i.e*. SP*_Usp45_*), which is the majority protein secreted by *L. lactis* ^26^. Indeed, this SP*_Usp45_* has been used to export many heterologous proteins in *L. lactis* ^27^. **pCWA**: to obtain a protein anchored to the bacterial wall, the gene of interest is fused to SP*^Usp45^* and the anchoring domain of the *S. pyogenes* M6 protein (CWA*_M6_*). This domain contains the necessary signals for wall anchoring ^28^. This figure was created with Biorender.com (accessed date: 9^th^ June 2022).

Currently, a wide range of constitutive or inducible expression systems have been described in *L. lactis*.^19,22,23^ Based on these studies, we have developed in our laboratory a system for the production-export of heterologous proteins in *L. lactis* using a very stable and well-characterized model secreted protein, *Staphylococcus aureus* nuclease (Nuc).^24^ This system is composed of a family of vectors that allow controlled targeting of protein expression inside the cell, secreted into the external environment or anchored to the cell-wall, pCYT, pSEC, and pCWA, respectively, under the control of the nisin-inducible promoter (P*_nisA_*)^25–28^ (Figure 2). In addition, these vectors are functional in a wide range of LAB and bifidobacteria, as will be shown below, and have been used successfully for the production of numerous heterologous proteins in *L. lactis* (Table 1).

**Table 1.** Some examples of heterologous protein production in *L. lactis* for vaccination purposes.

*L. lactis* strain	Expression system	Target pathogen	Heterologous protein	Immune response	Experimental model	Reference
*L. lactis* NZ3900	NICE: pNZ8149-SLS-F5 and pNZ8149-SLS-F5-OmpH plasmids	Enterotoxigenic *Escherichia coli*	SLS-F5-OmpH	Mucosal IgA and serum IgG and cellular immune response	BALB/c mice	^29^
*L. lactis* MG1363	Constitutive lactococcal promoter P23:^30^ pLZ12Km2P23:PilM1 (PilM1) and PilM1 strains containing introduced peptides plasmids	*Staphylococcus aureus*	D3(22–33)	Mucosal and serum IgG and IgA	BALB/c mice	^31^
*L. lactis* MG1363	pH-inducible expression system: pAMJ and pAMJ- rOmp16-IL2 plasmids	*Brucella melitensis*	Omp16-Human IL-2	Serum IgG	BALB/c mice	^32^
*L. lactis* MG1363	pH-inducible expression system: pAMJ2008-CagL	*Helicobacter pylori*	CagL	Serum IgG, IgA and fecal IgA	BALB/c mice	^33^
*L. lactis* FnBPA+:	pValac vector for DNA delivery	*Mycobacterium tuberculosis*	ESAT-6 and Ag85A	Colonic tissue IgA and cellular immune response	BALB/c mice	^34^
*L. lactis* NZ900	NICE: NZ8123-HPV16-optiE7 plasmid	Human Papillomavirus Type 16	HPV-16 E7 oncogene	Serum IgG and vaginal IgA	Human	^35^
*L. lactis* NZ9000	NICE: pNZ8124::sip plasmid	Group B *Streptococcus*	SIP	Systemic and mucosal IgG and IgA and cellular immune response	C57BL/6 mice	^36^
*L. lactis* MG1363	Constitutive lactococcal promoter P32:^30^ pMG36e-VP1 plasmid	Enterovirus 71	VP1	Serum IgG and fecal IgA	BALB/c mice	^37^
*L. lactis* NZ9000	NICE: pNZ8150-pgsA-HAsd plasmid	H5N1, H3N2 and H1N1Influenza A viruses	HAsd	Serum IgG and mucosal IgA	BALB/c mice	^38^
*L. lactis* MG1363	Constitutive lactococcal promoter P32:^30^ pMG36e-TSOL18 and pMG36e-SP-TSOL18 plasmids	*Taenia Solium*	SP-TSOL18	Serum IgG and mucosal IgA and cellular immune response	Kunming mice	^39^
*L. lactis* MG1363	pH-inducible expression system: pSS1 plasmid	*Plasmodium falciparum*	PfCSP4/38	Serum IgG	CD1 mice	^40^
*L. lactis* NZ9000	NICE: pNZ8121- Ppsp15-egfp and pNZ8121-egfp plasmids	*Leishmania major*	PpSP15	Th1 type immune response	BALB/c mice	^41^
*L. lactis* NZ3900	NICE: pNZ8149-SECF1S1 plasmid	*Bordetella pertussis*	F1S1	Serum IgG and mucosal IgA	BALB/c mice	^42^
*L. lactis* IL1403	Constitutive lactococcal promoter Ptuf:^43^ pILPtuf.nCoV.h plasmid	SARS-CoV-2 virus	RBD S1	Serum IgG and fecal IgA	BALB/c mice	^44^
*L. lactis* NZ9000	NICE: pGEM-VP6 plasmid	Rotaviruses	VP6	Serum IgG and IgA	BALB/c mice	^45^

## The Nisin Induced Controlled Expression (NICE) system

Nisin is a bacteriocin from *L. lactis* widely used as an antimicrobial substance in the food industry. Eleven adjacent chromosomal genes (nisABTCIPRKFEG) are encoded for nisin biosynthesis and immunity.^46^ The nisA gene corresponds to the structural gene for nisin and the nisRK genes constitute the two-component system responsible for the induction of other genes in the cluster. The study of nisin biosynthesis regulation has revealed the process by which extracellular nisin binds to the

NisK protein which autophosphorylates and becomes capable of activating the NisR regulator by phosphorylation. NisR then activates the transcription of the *nisABTCIP* and *nisFEG* operons. Characterization of the regulation of this system has led to the development of an inducible expression system in *L. lactis* using the nisA gene promoter and the nisRK genes.^47^ The gene of interest is cloned downstream of the PnisA promoter and the plasmid is introduced into a strain possessing the nisRK genes (*i.e. L. lactis* NZ9000). The addition of sub-inhibitory amounts of nisin in the culture medium triggers the expression of the gene of interest in proportion to the amount of nisin present.

This versatile system is now widely used to express heterologous proteins not only in *L. lactis* but also in other LAB.^47^ However, it requires the presence of regulatory genes (see above), which limits the choice of appropriate production conditions in both biotechnological and laboratory applications, and the induction of GM strain cultures prior to their use *in vivo*.

An alternative is the use of expression systems that require neither the presence of regulatory genes nor the pre-induction of the cultures prior to their use. In fact, in recent years several regulated expression systems have been described for LAB, in which gene expression can be controlled by an inducer, a repressor or by environmental factors, such as pH, sugar, temperature, or ion concentrations.^48^ In the following paragraphs, we will present two new systems developed by our group and collaborators in which we exploit both stress- and sugar-inducible systems for the production of heterologous proteins in *L. lactis*.

## The Stress-Inducible Controlled Expression (SICE) system

Heat-shock proteins are a conserved group of proteins (among which are the groESL and DnaKJ-GrpE chaperone complexes) synthesized in response to different stress stimuli such as heat-shock, low pH, UV irradiation, or salts stress. The SICE system is based on the use of the groESL heat shock protein operon promoter from *L. lactis*. This episomal system is composed of a vector that carries an expression cassette under the transcriptional control of a stress-inducible promoter.^49^ In this system, the expression of the protein of interest is induced after administration to the host, since the GM bacterium finds different conditions than those of the culture and suffers different types of stress. Heat stress due to the body temperature of the host higher than the optimal growth temperature of the bacteria; and in the case of oral administration, the acid stress during passage through the stomach added to biliary stress in the duodenum are examples of stresses able to induce expression in this system, and allow the *in situ* production of the molecule of interest. The main advantage of this system is that it does not require the presence of regulatory genes or the induction in cultures before use.^50,51^

## Xylose-Inducible Expression System (XIES)

This is a controlled production system that allows target heterologous proteins to cytoplasm or extracellular medium. It was described for *L. lactis* NCDO2118 and uses a xylose-inducible lactococcal promoter, PxylT^52^(Miyoshi, 2004). The system was tested using the *Staphylococcus aureus* nuclease gene (nuc) to produce cytoplasmic and secreted proteins when they are fused or not to the SP*_Usp45_* (see above Figure 2). Nuc is considered a good reporter protein because its activity is very easily detected *in vivo* by a staining test on bacterial colonies, in liquid culture or extracted from the digestive tract. This expression system can be switched on or off by adding either xylose or glucose, respectively, and was also used for the production of proteins of interest in *L. lactis*.^53,54^

## *Other vectors and cellular factors for improved production and secretion of heterologous proteins in* L. lactis

In addition, another expression vector was further developed containing a synthetic propeptide (LEISSTCDA), which has been identified as an enhancer of production and secretion in *L. lactis*.^55,56^ Moreover, considering host factors affecting production-secretion, the *ybdD* gene was identified; and the inactivation of this gene resulted in overproduction of only secreted proteins by a mechanism not yet elucidated. *L. lactis* secretion machinery was also complemented with SecDF from *Bacillus subtilis* and it was observed that the resulting strain improved production and secretion.^57^ Finally, we have also developed in our team to complete our toolbox of heterologous protein expression in L. lactis, a strain that produces neither the unique extracellular protease HtrA^58^ nor the major intracellular protease ClpP.^59^ This double mutant strain allows a controlled and stable production of different heterologous proteins that are otherwise highly degraded in the wild-type strain.^60^

In summary, numerous studies currently support the use of GM strains of *L. lactis* to induce both mucosal and systemic immune response.^19,22^ In this context, the first attempt to evaluate the potential of *L. lactis* as a mucosal vaccine, more than 30 years ago, was performed with a GM strain of this bacterium producing an anchored form of the protective antigen (PAc) of *Streptococcus* (*S*.) *mutans*.^61^ This study shows for the first time that *L. lactis* can be used as a live mucosal vector to efficiently deliver an antigen to the immune system. Then, Wells et al. reported that the use of a live GM *L. lactis* strain producing tetanus toxin fragment C (TTFC) as a model antigen was able to protect mice against a lethal challenge with tetanus toxin after subcutaneous administration of this strain.^62^ Later, the same group evaluated the effect of oral or nasal administration of GM TTFC-producing strains of lactococci in mice.^63,64^ Oral immunization in mice with these GM lactococci results in a lower humoral response (i.e., TTFC-specific serum IgG and mucosal IgA antibodies) than intranasal administration, but the measured protective efficacy (challenge with tetanus toxin) was the same.

Following these pioneering studies, several works have evaluated the expression of numerous heterologous proteins of viral, bacterial, or eukaryotic origin in *L. lactis* (Table 1). The immunogenicity of the resulting GM strains was also evaluated in animal models with very promising results. In particular, our team succeeded (as mentioned above) in producing about 40 heterologous proteins in *L. lactis* (Table 1). Since our main goal was to induce an immune response at the host mucosal level, our efforts were mainly focused on the presentation of medically relevant proteins such as cytokines, antigens, allergens, and antioxidants by LAB.

Altogether, our results together with findings from other teams confirm the potential of GM strains of *L. lactis* as live mucosal vectors of proteins of health interest and open the way for such GM LAB strains to be approved to bring new solutions in the future for disease prevention and treatment.

## Lactobacilli as a live delivery vector

Unlike *L. lactis*, some lactobacilli species can colonize certain regions of the mucosa and induce a local immune response, which is an advantage in vaccine development. Furthermore, the possible use of certain probiotic strains of lactobacilli as a mucosal delivery vehicle is an additional advantage.^65^ However, the biodiversity of this genus makes its use as a vector for live delivery vector more complex than that of *L. lactis*, where only one strain (MG1363) has been used. Indeed, this genus is widespread and contains more than 60 species that differ in their biochemical, ecological, and immunological properties. However, the ability of the genus *Lactobacillus* to produce antigens has also been demonstrated. Nonetheless, the ability of the *Lactobacillus* genus to produce antigens has also been demonstrated; in fact, different studies have shown that vaccines based on lactobacilli are capable of inducing a strong humoral and cellular immune response in both blood and mucosa when administered orally or intranasally (Yu et al., 2013).

## Production of heterologous proteins in *Lactobacillus spp.*

In the late 1990s and early 2000s, several laboratories reported the use of GM strains of lactobacilli as live vectors to deliver proteins of medical interest to the mucosal surface.^23,65^ Thus, we decided to transfer all our knowledge on the production of heterologous proteins acquired in *L. lactis* to *Lactobacillus* strains (more specifically to *Lactobacillus casei* and *Lactiplantibacillus plantarum* strains), which present the double interest of transiting slower in the digestive tract and display interesting adjuvant properties.

## The Lactobacilli In Vivo Expression (LIVE) system

This system consists of a plasmid that allows the production and secretion of inducible heterologous proteins in vivo by several strains of lactobacilli.^66^ Indeed, for the construction of this plasmid, called pLB210, the stress-inducible promoter P*_lp_07755_* from *L. plantarum* WCFS1 was cloned into the plasmid pLB141^48^ and then used to transform the lactobacillus strains. Thus, it was confirmed that this LIVE system is an *in vivo*-regulated expression system that is mainly induced by stress conditions such as exposure to high temperature or high concentrations of bile salts.^66^

The LIVE system was used for the expression of the anti-inflammatory cytokine IL-10 as a therapy against intestinal inflammation. To this end, a DNA fragment coding for murine IL-10 was cloned into the plasmid pLB210 and used to transform different species of lactobacilli. The GM bacterium was then evaluated in a murine model of IBD and the results demonstrated the efficacy of the LIVE system to produce and deliver the therapeutic molecule in the mucosal surface, since an increase in the ratio of anti-inflammatory/pro-inflammatory cytokines was evidenced at intestinal level.^66^

In addition, the LIVE system was also evaluated in an immunization model against a bacterial pathogen, evaluating the antigen *GbpB* production and stimulation of the immune response against a cariogenic strain *S. mutans* with the aim of combating dental caries. Similarly, the *GbpB* gene from *Streptococcus mutans* was cloned into the plasmid pLB210, which was used to transform the lactobacillus strain. The GM strain expressing the *GbpB* antigen was used to immunize mice and the results showed that the LIVE system allowed the expression of the antigen and stimulation of the host’s immune response.^66–68^

This system has advantages over to expression systems based on preinduction prior to oral administration or genetic mutation on chromosomal bacterial DNA.

## Production of heterologous proteins in *Lactobacillus spp*Similar

To *L. lactis*, several works have reported the expression of a variety of heterologous proteins of viral, bacterial, or eukaryotic origin in lactobacilli (Table 2). For instance, the use of GM lactobacilli to produce heterologous proteins and to develop a new generation of mucosal vaccines was first proposed in the early 1990s, but most of our current knowledge on their use as a live vaccine was obtained with the model antigen TTFC.^81,82^ Indeed, taking into account the positive results obtained with the expression of tetanus toxin fragment C (TTFC) in *L. lactis*, similar tools were applied in lactobacilli. In the first study, cell extracts of the GM *Lactobacillus casei* expressing TTFC were used to parenterally immunize mice and to evaluate the immune response. In addition, in another study, GM *Lactobacillus* strains producing TTFC in three different cellular locations: cytoplasm, secreted, or anchored to the cell-surface were used to immunize mice by subcutaneous, intranasal, and intragastrical routes. In both studies, the induction of the immune response against TTFC was observed.^83,84^

**Table 2.** Some examples of heterologous protein production in lactobacilli for vaccination purposes.

Lactobacilli strain	Expression system	Target infective agent	Heterologous protein	Immune response	Experimental model	Reference
*L. plantarum* NC8	pVALAC vector for DNA delivery	*Trinchinella spiralis*	CPF1 and IL-4	Serum IgG, mucosal IgA and cellular immune response	BALB/c mice	^69^
*L. casei* ATCC393	Constitutive expression pPGT7g10-PPT vector	*Clostridium perfringens*	α, έ, β1, and β2 toxoids	Serum IgG, mucosal IgA and cellular immune response	BALB/c mice	^70^
*L. plantarum* HQ542228	Constitutive lactococcal promoter P32^30^	J Avian Leukosis Virus	gp85	Serum IgG and mucosal IgA	Hy-Line Brown layer chickens	^71^
*L. plantarum* NC8	Inducible pSIP409 vector	*Eimeria tenella*	EtMic2	Serum IgG and mucosal IgA	1-day-old broilers	^72^
*L. plantarum* NC8	Inducible pSIP409 vector	Rabies virus	G gene-DCpep	Serum IgG	BALB/c mice	^73^
*L. casei* ATCC393	Constitutive expression pPGT7g10-PPT vector	Rabbit hemorrhagic disease virus	VP60(VP1)	Serum IgG and mucosal IgA	two-month-old rabbits	^74^
*L. plantarum* WCFS1		*Mycobacterium tuberculosis*	Ag85B-ESAT-6	Cellular immune response	C57BL/6 mice	^75^
*L. plantarum* NC8	Inducible pSIP409-pgsA vector	Group A rotavirus	VP7-DCpep	Serum IgG, mucosal IgA and cellular immune response	BALB/c mice	^76^
*L. plantarum* WXD234	Inducible pNZ8148 vector	*Staphylococcus aureus*	HlaH35L	Serum IgG, mucosal IgA and cellular immune response	C57BL/6 mice	^77^
*L. plantarum* NC8	pVALAC vector for DNA delivery	*Eimeria tenella*	EtMIC2 and IL-18	Serum and mucosal IgA and cellular immune response	Newly hatched broiler chickens	^78^
*L. casei* CICC 6105	pLA vector	Enterotoxigenic *Escherichia Coli*	987P	Serum IgG, mucosal IgA and cellular immune response	BALB/c mice	^79^
*L. plantarum* CGMCC 1.557	Inducible pSIP411 vector	SARS-CoV-2	RBD	Mucosal IgA	BALB/c mice	^80^

Our team also explored the immunogenicity of a GM strain of *L. plantarum* producing the HPV-16 E7 antigen in animal models with very promising results.^85^ We have also developed GM *L. casei* strains producing antioxidant enzymes such as catalases or superoxide dismutase. The beneficial effects of oral administration of lactobacilli strains with antioxidant properties in a murine model of inflammation were evaluated in order to reproduce Inflammatory Bowel Disease (IBD) syndromes. IBD is a disease caused by abnormal inflammation of the intestinal tract that leads to gastrointestinal dysfunction. These diseases, often disabling and of long duration, are characterized by an excess of active oxygenated derivatives, accompanied by decreased capacities of the antioxidant systems as well as an imbalance between pro- and anti-inflammatory cytokines. First, a manganese-dependent catalase (MnKaT) from *L. plantarum*^86^ was expressed in *L. casei* BL23 and its anti-inflammatory effect was evaluated in a mouse model of colitis induced by DSS (Dextran Sulfate Sodium). The results showed that a daily oral administration of both wild-type and MnKat catalase-producing *L. casei* strains significantly limited inflammation in the cecum and colon, in contrast to control mice treated with PBS alone, in which diarrhea and mucosal lesions were observed.^86^ Then, in order to improve the antioxidant potential of these strains, the *L. lactis soda* gene

was expressed in the *L. casei* BL23 strain, and tested for the manganese superoxide dismutase (MnSOD) activity as well as an antioxidant and anti-inflammatory effect.^87^

In another study, Chang *et al*. constructed a GM strain of *L. jensenii*, a commensal vaginal bacterium, to express and secrete a domain of the human immunodeficiency virus (HIV) gp120 binding protein, CD4, and demonstrated that co-incubation of this GM bacterium with an HIV virus carrying the luciferase reporter gene (*e.g*., HIV-1HxB2) results in a significant decrease in the entry of this virus into HeLa cells (expressing the CD4-CXCR4-CCR5 receptor) *in vitro*.^88^

It has also been reported that the immune response obtained is related to the different origins of the strains. Two GM strains LA4356-pH and DLD17-pH that express the foreign HPAI virus protein hemaglutinin 1 (HA1) were constructed and orally administered to mice in which mucosal and systemic immune responses were assessed. It was observed that both GM strains were able to increase the anti-HA1 IgA antibodies’ level in the mucosa and the anti-HA1 IgG level in serum. However, DLD17-pH induced a mucosal immune response in both the digestive and respiratory tracts while LA4356-pH only in the digestive tract. This difference was attributed to the different origin of the strains, since DLD17 was isolated from chicken gut and therefore DLD17-pH could better adapt and survive in the intestine and persistently stimulate the immune response, while LA4356 was isolated from human pharynx and therefore the adhesion to intestine of this strain could be weak, resulting in a lower mucosal immune response.^89^ Later, studies showed that in addition to the origin of the strain used for the delivery to the host, other variables can influence the immune response obtained such as the different expression systems used that can affect antigen expression levels^90^ or even the chosen immunization route.^91^ The mode of presentation of the antigen (cytoplasmic, secreted, or associated with the cell surface) is also a factor that influences the immune response.^92^

In conclusion, different studies have shown that it is possible to enhance the immune response induced by lactobacilli vaccine vectors against pathogens. Furthermore, besides their application as vaccines, lactobacilli can also be used to deliver anti-infective molecules or antimicrobial products *in situ*. An example is the use of GM strains of lactobacilli to prevent dental caries in an animal model.^93^

## *Bifidobacterium spp*. as live mucosal delivery vector

The bifidobacteria genus is included within the Actinobacteria phylum and constitutes one of the dominant populations of the human intestinal microbiota, especially in infants. Some strains of bifidobacteria showed beneficial effects in the prevention and treatment of diseases and are therefore considered probiotic microorganisms.^94^ *Bifidobacterium* spp. has advantages over lactobacilli and lactococci for its use as live vector for protein delivery. Certain strains have been shown to reside longer in the digestive tract than strains of lactobacilli and lacotococci (which do not colonize the intestine). On the other hand, bifidobacteria have low resistance to antibiotics (intrinsic and acquired), being safe for use in humans.^95^ There are only few studies about the use of bifidobacteria as vectors for the delivery of heterologous proteins due to considering them complex to manipulate genetically and only some strains have been used for this purpose. GM bifidobacteria has been used for cancer therapy,^96,97^ and as live vaccine to express antigens of pathogenic bacteria.^98–100^

## Bifidobacteria Expression SysTem (BEST)

This system, similar to NICE and SICE systems for lactococci and LIVE system for lactobacilli, is a controlled expression system for the delivery of heterologous proteins in bifodobacteria. This system has three constituents: a broad-host range plasmid pWV01, a stress inducible dnaK promoter from *Bifidobacterium* (*B*.) *longum* dnaK operon, and two different signal peptides (SPs): one issued from *L. lactis* (SP_Exp4_) and one from *B. longum* (SP_BL1181_).^95^

The BEST system was used to express the murine anti-inflammatory cytokine IL-10 in *B. bifidum*. For the construction of the plasmid, the promoter of the DnaK operon of *B. longum* was cloned into the plasmid pLB270 resulting in pBESTExp4:Nuc. The P*_nisA_* promoter was replaced by P*_DnaK_* since the *dnak* operon encodes for heat-shock proteins and the transcription of these proteins is increased by exposure to bile salts and different pH. The *nuc* genes were replaced by the murine IL-10 gene obtaining the plasmid pBESTExp4:IL-10 which was established in *B. bifidum* BS42. In order to increase the levels of secreted IL-10, SP*_Exp4_* from *L. lactis* was replaced by SP*_BL1181_* from *B. longum. The in vivo* results showed that the GM bacteria were capable of delivering IL-10 at intestinal level with better results for *B. bifidum* harboring pBEST*_BL1181_*:IL10 plasmid than for *B. bifidum* harboring pBEST*_Exp4_*:IL-10 plasmid, and this cytokine was capable of exerting its anti-inflammatory effect in an IBD murine model.^95^

## Applications of GM LAB in health

**Virus infections**. HPV-16 is one of the viruses with oncogenic potential found (along with type 18) in more than 90% of cervical cancers (300,000 deaths per year worldwide).^101,102^ Current strategies to prevent or treat the infection with this virus are promising but expensive, limiting their use in developing countries where there are about 80% of HPV-related cancer deaths. Prophylactic vaccines based on virus-like particles (VLPs) have recently induced significant reductions in HPV-16 and HPV-18 infections and associated cancers in human clinical trials. *L. lactis* was GM to deliver two proteins: ***i)*** the HPV-16 E7 antigen, a protein consistently found in carcinomas caused by HPV infections and one of the candidate antigens for the development of anti-HPV therapy, and ***ii)*** interleukin-12 (IL-12), a stimulatory molecule of the cellular immune response during infections.^103^

A GM strain of *L. lactis* was constructed to secret the E7 antigen, which is described as a very labile protein.^104^ E7 was also targeted to the expression in three different locations (*i.e*. cytoplasm, wall and extracellular medium), and it was demonstrated an E7-specific immune response in mice following nasal administration with the three GM strains expressing E7. It was also observed in an increased immunogenicity of the anchored form of E7.^105^

Recently, GM LAB strains have been also constructed to fight against respiratory virus infection. Severe Acute Respiratory Syndrome Coronavirus −2 (SARS-CoV-2) is a novel member of beta-coronavirus that causes a severe respiratory syndrome called coronavirus disease-19 (COVID-19). The COVID-19 pandemic was declared by the WHO due to the rapid spread of the virus worldwide, which was associated with high morbidity and mortality.^106^ Vaccines and therapeutic agents are still being studied with the aim of preventing the spread of the virus and achieving mass immunity in order to restore social and economic activities.^107^ A mucosal vaccine was developed using a GL *L. plantarum* expressing on its surface the receptor-binding domain (RBD) of the SARS-CoV-2 spike protein. The immune response evaluated in mice after intranasal administration of the GM strain showed an induction of the humoral immune response at respiratory and gastrointestinal mucosal levels.^80^

In addition, oral immunization of mice with cell extracts from a GM strain of *L. lactis* expressing SARS-CoV-2 Spike Protein RBD S1 subunit was also studied. The results showed the induction of the mucosal and systemic immune response with the production of specific antibodies, showing that this strategy could be used to develop oral viral vaccines.^44^

Other strains of LAB have also been studied as live vaccines against other respiratory viruses. A GM strain of *Enterococcus faecium* L3 expressing either HA2 hemagglutinin subunit of H_1_N_1_ influenza virus or its conserved part, long alpha helix (LAH) antigen, in combination with four conserved extracellular domain of the matrix protein 2 (M2e) epitopes was used to generate two mucosal vaccines against influenza virus. Oral immunization of mice demonstrated the induction of systemic humoral immune response.^108^ The same strain expressing the antigens of chimeric protein PSPF (*Pneumococcus* Surface Proteins and Flagellin) was previously studied as a mucosal vaccine against the *S. pneumoniae s*howing the versatility of the probiotic vaccine against a range of respiratory pathogens.^109^

Regarding bifidobacteria, a study reported the cloning and expression of enterovirus 71 capsid protein 1 (EV71-VP1) in a strain of *B. pseudocatenulatum* M115 showing the possibility of using bifidobacteria for the expression of genes encoding virulence factors.^110^

GM LAB have also been studied as vectors for the production of heterologous proteins of animal viruses. Porcine epidemic diarrhea virus (PEDV) is a member of alphacoronavirus that causes an intestinal disease which have caused economic losses around the world.^111^ A GM strain of *L. casei* has been used to express N antigen protein of PEDV, and administered oral and intranasally to pregnant sow and mice. This strain was able to both induce mucosal and systemic immune response.^112^ The same strain was GM to express the protein S of the PEDV virus and the GM *L. casei* was administered to mice observing an induction of cellular, humoral, and mucosal immunity.^113^ A bivalent oral vaccine against PDEV and TGEV (porcine transmissible gastroenteritis virus) was also developed using a strain of *L. casei* which was GM to express TGEV S protein D antigen and PEDV S protein-neutralizing antigen epitope region COE as immunogens. Immunized mice showed an induction of humoral and mucosal immune response against TGEV and PDEV, evidencing the potential of this vaccine to prevent both infections.^114^ In addition, other systems have been developed for the expression of proteins using *L. lactis* as a vector for the prevention of avian influenza virus infection and infection bursal disease in chicken.^115,116^

**Bacterial infections**. Gastroenteritis of bacterial origin is a disease characterized mainly by episodes of diarrhea caused by pathogens such as *E. coli, Vibrio cholerae, Campylobacter* spp., *Salmonella* spp., *Shigella, Aeromonas* spp., and *Yersinia enterocolitica*, among others.^117^ Different studies showed the potential of using LAB as mucosal vaccines against these intestinal pathogens. In these senses, a GM *L. lactis* expressing HCP (Hemolysin co-regulated protein) of *Campylobacter jejuni* T6SS was administered to mice, and the results showed the induction of specific neutralizing antibodies and the prevention of pathogen colonization.^118^ In addition, a GM *L. lactis* expressing the binding domain of heat-labile toxin (LBT) from enterotoxigenic *E. coli* (ETEC) was used to immunize rabbits and was able to induce the production of antibodies at the intestinal mucosa level.^119^

LAB have also been studied as mucosal vaccines against respiratory pathogens. A vaccine against tuberculosis was developed by constructing a GM strain of *L. Lactis* expressing two antigens of *Mycobacterium tuberculosis*: Early Secreted Antigenic Target (ESAT-6) and the antigen 85 complex (Ag85A). The GM strain was used to immunize mice in which induced humoral and cellular immune responses.^120^ In another study, an oral vaccine against *S. aureus* was developed. For this purpose, a strain of lactobacilli synthesizing *S. aureus* nontoxic mutated-hemolysins (HlaH35L) was constructed, and the GM strain was able to induce the mucosal immune response in mice and protected against pulmonary and skin infection.^77^ This study demonstrated the potential of lactobacilli to be used as a delivery vector in the development of oral vaccines against bacterial pathogens.

## Parasites infections

Trichinellosis is a disease caused by the parasitic nematode *Trichinella*. Infection in humans is caused by the consumption of larvae present in raw or undercooked meat.^121^ A vaccine against *Trichinella* (*T*.) *spiralis* was developed using a strain of *L. plantarum* coexpressing the *T. spiralis* cathepsin F-like protease 1 gene (TsCPF1) and murine IL-4 (mIL-4). After immunization, mice showed the production of specific antibodies which protected against *T. spiralis* infection.^69^

Leishmaniasis is a disease caused by more than 30 species of the *Leishmania* parasite and is transmitted by the female sandfly vector to humans.^122^ Different alternatives have been evaluated with the aim of developing a live oral vaccine for this human parasite. A strain of *L. lactis* co- expressing the protective *Leishmania* homologue of activated C kinase (LACK) and mouse IL-12 induced an antigen-specific mucosal immune response in protected mice.^123^ Another study used a GM strain of *L. lactis* to express the protein PpSP15 an immunogenic component of saliva from the sand fly *Phlebotomus papatasi*. The strain was evaluated to immunize mice and it was described the induction of a strong immune response with a long-term protection against *Leishmania major*.^41^

Malaria is a disease caused by different species of the *Plasmodium* parasite and transmitted by the female *Anopheles* mosquito.^124^ Different vaccines have been studied with the aim of producing specific antibodies against proteins expressed during the development of the parasite in the mosquito. In this sense, *L lactis* has been used to express the cysteine-rich Pfs48/45 protein, exposed on the surface of sexual stages of the parasite^125,126^ or the Circumsporozoite protein (PfCSP), a sporozoite surface protein essential for its development in the mosquito and cell invasion in the mammalian host.^40^ The results showed the induction of high levels of functional antibodies in rodents. The expression in *L. lactis* of the fusion protein Pfs230-Pfs48/45 was also studied and the final product elicited high levels of functional antibodies in mice.^127^

Chagas disease is an infectious disease caused by the parasite *Trypanoma cruzi*.^128^ A mucosal vaccine was designed using a GM *L. lactis* co-producing the antigen (a fragment of the trans-sialidase (TScf) enzyme from the *Trypanosoma cruzi* parasite) and the mucosal adjuvant 3’ 5’- cyclic di adenosine monophosphate(c-di-AMP). Immunization of mice with the engineered bacteria induced a specific immune response against the antigen.^129^

In addition, other vaccines have been developed using *L. lactis* as a vector to deliver antigens from other parasites such as *Taenia solium* and *Echinococcus granulosus*; these strains were able to stimulate in mice the immune response against these diseases that affect both animals and humans.^39,130^

**Cancer**. The cytokine IL-12 has already successfully used in immunotherapy and cancer therapy. A GM strain of *L. lactis* secreting a native heterodimeric form of IL-12 was constructed. The biological activity of IL-12 produced by *L. lactis* was then confirmed *in vitro* in mouse spleen cells and *in vivo* by intranasal administration to mice and as adjuvant by combining them with the *L. lactis* strain producing the anchored form of the E7 antigen.^131^ In order to evaluate the preventive and curative capabilities of the combination of these two lactococci, a mouse model was developed in which tumors were induced by subcutaneous implantation of tumor cells expressing HPV-16 antigen E7 (TC-1), and their progression was measured following intranasal administration of the strains.^132^ The preventive and curative effects of intranasal co-administration of E7- and IL-12-producing lactococcal strains in mice were evaluated on TC-1 tumor development. The results demonstrated that preventive administration of lactococci, before tumor cell injection, induced the absence of tumor development in 50% of the immunized animals. In addition, a significant adjuvant effect of IL-12 co-delivered with the E7 antigen was found; in the absence of the IL-12 producing strain, the absence of tumors was observed in only 25% of immunized mice. Moreover, mice immunized with LL-E7 and LL-IL12 were able to resist a second challenge (2 months after the first immunization) suggesting that the induced immunity is durable.^132^ Therapeutic use of these strains in mice with already implanted tumors resulted in complete regression of tumors in 35% of treated animals. These anti-tumor effects were the consequence of a cytotoxic response dependent on CD4+ and CD8 + T lymphocytes. These results in mice constitute the first evidence of a preventive and curative effect against cervical cancer by mucosal vaccination with GM lactococcal strains.

## Allergy, Inflammation, and autoimmune disease

Allergic disease is a chronic inflammatory disorder characterized by a dysregulated immune response to allergens. The most common allergies include allergic asthma, allergic rhinitis, atopic dermatitis, and food allergies. In most cases, patients do not respond to conventional treatments. In this sense, LAB-based mucosal vaccines are an attractive option for the prevention and treatment of allergic diseases.^133–136^ A mucosal vaccine based on *L. lactis* expressing major dust mite allergen Der p2 was developed, and its prophylactic effect was evaluated in a Der p2-sensitized mouse model. The results showed that the GM LAB was able to prevent the development of allergen-induced airway inflammation primarily by the induction of specific mucosal immune tolerance with reduction of inflammatory parameters. *L. lactis* has been also used as a vector to express Ara h 2.02 (one of the two isoforms of the Ara h 2 major peanut allergen) and administered to allergen-sensitized and -challenged mice. Animals that received the GM bacteria showed an alleviation of the Th2-associated responses.

*L. lactis* strains have also been used to treat other inflammatory pathologies. A GM strain was evaluated for secreting bioactive hemeoxygenase-1 (HO-1) in a model of hyperoxia-induced lung injury in rat pups. It was observed that the intranasal administration of the GM bacteria was able to prevent pulmonary inflammation through the attenuation of inflammatory parameters ^136^. *L. lactis* expressing therapeutic proteins has been studied in different models of intestinal inflammation. One study used a GM strain to deliver IL-10 in a mouse model of chronic irritable bowel syndrome and it was demonstrated a beneficial effect.^137^ In another study, *L. lactis* was also used to express IL-27, an immunosuppressive cytokine, and attenuation of colitis in mice was observed.^138^ A strain of *L. lactis* engineered to express human pancreatitis-associated protein I (PAP) was also used to prevent intestinal mucositis in mice.^139^

The anti-inflammatory effect associated with the administration of milks fermented by strains of *L. lactis* that express IL-10 under the control of the XIES was evaluated using a TNBS-induced colitis murine model.^140^ Milks fermented by strains producing IL-10 in the cytoplasm (Cyt strain) or secreted (Sec strain) showed decreased inflammation in their large intestines with a regulated immune response. In another study, considering that reactive oxygen species are involved in the intestinal inflammation, *L. casei* BL23 strains producing either catalase (CAT) or superoxide dismutase (SOD) were evaluated in mice before and after intrarectal administration of TNBS. These strains were associated with faster recovery of initial weight loss, and decrease of intestinal inflammation.^141,142^

Type 1 diabetes is a chronic autoimmune disease characterized by a destruction of the insulin-producing β cells of the pancreas due to attack by autoreactive T cells resulting in hyperglycemia. *L. lactis* has been studied as a vehicle for oral vaccines in the treatment of different autoimmune diseases. Oral immunization with a GM strain of *L. lactis* expressing the heat shock protein 65 and tandemly repeated IA2P2 (HSP65-6IA2P2) in mice was studied. It was observed that the GM strain was capable of efficiently delivering the antigen at the mucosal level, inducing immunotolerance and preventing the appearance of type 1 diabetes in animals.^143^ A genetically modified *L. lactis* strain was also used as a strategy to administer proinsulin and IL-10 combined with low dose of anti-CD3 (aCD3) and it was observed a restoration of β-cell tolerance and glucose homeostasis in diabetic mice.^144^ In addition, a GM strain of *L. lactis* expressing IL-4 and IL-10 was able to protect against type 1 diabetes in mice by preventing hyperglycemia and reducing pancreatic cell destruction.^145^

Multiple sclerosis is an autoimmune neurological disease that results in destruction of the central nervous system white matter.^146^ Current treatments are only useful to reduce symptoms and slow the progression of the disease, and can even have severe adverse effects; therefore, new therapies are being studied. *L. lactis* has been used as vector for the expression of heat shock protein (Hsp65) and specific epitopes of the three main myelin proteins (myelin oligodendrocyte glycoprotein MOG, myelin basic protein MBP, and proteolipid protein PLP). GM LAB were evaluated in models of experimental autoimmune encephalomyelitis (EAE) in mice and rats, respectively, and were able to prevent the development of the disease or reduce the clinical symptoms.^147–149^

Rheumatoid arthritis is a chronic autoimmune disease that mainly affects cartilage and bone.^150^ Two different studies used a strain of *L. lactis* engineered to deliver IL-5 and Hsp-65, respectively, and the GM strain was able to attenuate or prevent collagen-induced arthritis in mice.^151,152^

In addition, LAB have been used to express antigenic proteins for the treatment or prevention of other autoimmune diseases such as Sjogren’s syndrome, a disorder that mainly affects the lacrimal and salivary glands. A strain of *L. lactis* genetically modified to express enterotoxigenic *E. coli* colonization factor antigen I (CFA/I) was evaluated in a murine model of Sjogren’s syndrome and the ability of the GM strain to reduce the progression of the disease was demonstrated.^153^

## Metabolic disorder

Obesity is a global public health problem and treatments to reduce this problem have a very high therapeutic potential. A GM lactococci that produce human leptin, a hormone produced mainly by the adipocyte, which informs the brain of the state of adipose reserves was developed. In obese mice (*ob*/*ob*), leptin deficiency leads to massive obesity. The aim of the work was to measure the effects of nasal administration of a strain of *L. lactis* secreting human leptin in ob/ob mice. First, a strain of *L. lactis* that efficiently secreted biologically active human leptin was constructed. Then, it was determined whether intra-nasal administration of LL-Lep could inhibit food intake and weight gain in ob/ob mice. It was observed that daily administration of this GM strain of *L. lactis* to these obese mice significantly reduced weight gain and food intake.^154^ These results demonstrate that leptin is produced in an active form by *L. lactis* and that this strain can be successfully used to regulate body weight and food intake.

## Current status of the use of GM LAB and Bifidobacteria in clinical trials

Although different groups have reported the use of GM LAB and Bifidobacteria to treat different diseases in humans and animals, few studies have advanced to clinical trials. This is certainly due to the fact that it is necessary to integrate certain important features in the successful development of such GMM as live biotherapeutics, such as transient presence in the host gut (e.g. humans), a biocontainment strategy, biomarkers of activity and compliance with FDA (Food and Drug Administration, United States) requirements.

One of the few human clinical trials properly conducted considering all these factors were by Steidler et al. Indeed, data obtained by this group in a Phase 1 clinical trial conducted with a human IL-10-secreting GM strain of *L. lactis*^155^ showed that the containment strategy used to construct the strain^156^ was not only safe and effective but also that mucosal delivery of IL-10 by a GMM is feasible in humans.^157^ In addition, a Phase 2a clinical trial in patients suffering from Crohn’s disease (a type of IBD) confirmed that the primary endpoints of the study were met with this GM *L. lactis* strain expressing IL-10: *i.e*., safety and tolerability of the GM strain, environmental containment of the GM organism, and assessment of strain-associated biomarkers. Unfortunately, concerning the disease progression endpoints, the clinical results did not reveal a statistically significant difference in mucosal healing compared to the placebo group (https://clinicaltrials.gov/ct2/show/NCT00729872). However, a new study from the same group demonstrated in another Phase 1b clinical trial that the use of a strain of *L. lactis* expressing another therapeutic molecule (*i.e*., human trefoil factor 1,^158^) showed that this GMM was effective in treating oral mucositis, a major inflammation and ulceration of the membranes lining the oral cavity, throat and esophagus that is among the most commonly reported adverse events associated with cancer chemotherapy. Interestingly, preliminary data demonstrated the positive efficacy of this GM strain against oral mucositis in 25 patients compared to placebo.^159^ Finally, our group is currently developing a biosafety strategy to use a GMM expressing human elafin in human clinical studies (Ref.^160^ and unpublished data).

## Discussion and conclusions

In the last years, the interest in the use of LAB and bifidobacteria to deliver molecules of interest has increased considerably, resulting in significant advances that are gathered in this review. In spite of this progress, many questions remain unanswered, notably concerning the immune response generated in the host by the native antigens of the LAB used as a vector or the mode of oral or intranasal administration. Currently, the latter seems to be the most adapted to induce a good immune response at the systemic and mucosal levels, but the health aspect of these administrations remains to be ensured.

Therapeutic applications have evolved in such a way that we can reasonably envisage the use of GM LAB in the treatment of human pathologies in the coming years. Biological containment systems have been developed to prevent the dissemination of these GM LAB. This strategy has allowed the implementation of some Phase I and Phase II clinical trials (see above) which is an essential step in the future use of these very promising tools.
